# Book Reviews

**Published:** 1874-03-15

**Authors:** 


### Book Reviews.

AN Universal Formulary; containing the Methods of Preparing and Administering Officinal and other Medicines; the whole adapted to Physicians and Pharmaceutists. By R. Eglesfeld Griffith, M.D. Third edition. Carefully revised and much enlarged, by John M. Maisch, Phar. D., Professor of Materia Medica and Botany in the Philadelphia College of Pharmacy. With illustrations. 8vo.; pp. 779. Philadelphia: Henry C. Lea, 1874.

The third edition of this excellent Formulary, familiar for many years to both physicians and druggists, appears with a new name impressed upon its title page. It is that of the editor of the *American Journal of Pharmacy*, and the Permanent Secretary of the American Pharmaceutical Association; and it suggests to those who have recognized his ability as displayed in these positions, a thorough and efficient discharge of the editorial duties here assumed.

In this edition we are pleased to observe the retention of the alphabetical arrangement of the names of remedies according to their pharmaceutical titles in the United States Pharmacopœia, as it facilitates the work of reference: though copious indices are also appended. We find here, also, revised tables of specific gravities, hydrometrical equivalents, etc., as well as formulas for all new remedies of acknowledged value, while we miss many of the old ones, which deserved to be rejected on account of their worthlessness.

This Formulary has already proved itself acceptable to the medical profession; and we do not hesitate to say that the third edition is much improved and of greater practical value, in consequence of the careful revision of Professor Maisch.

